# Fenofibrate’s impact on cardiovascular risk in patients with diabetes: a nationwide propensity-score matched cohort study

**DOI:** 10.1186/s12933-024-02353-5

**Published:** 2024-07-18

**Authors:** Sangmo Hong, Kyung-Soo Kim, Kyungdo Han, Cheol-Young Park

**Affiliations:** 1 Department of Internal Medicine, Guri Hospital, College of Medicine, Hanyang University, Seoul, Republic of Korea; 2 Department of Internal Medicine, CHA Bundang Medical Center, CHA University School of Medicine, Seongnam, Republic of Korea; 3 Department of Statistics and Actuarial Science, Soongsil University, Seoul, Republic of Korea; 4 Division of Endocrinology and Metabolism, Department of Internal Medicine, Sungkyunkwan University School of Medicine, Kangbuk Samsung Hospital, Pyung-Dong, Jongro-Gu, (03181), Seoul, Republic of Korea

**Keywords:** Cardiovascular diseases, Diabetes, Fenofibrate, Mortality, Statin, Triglycerides

## Abstract

**Background:**

The beneficial effects of fenofibrate on atherosclerotic cardiovascular disease (ASCVD) outcomes in patients with diabetes and statin treatment are unclear. We investigated the effects of fenofibrate on all-cause mortality and ASCVD in patients with diabetes, high triglyceride (TG) levels and statin treatment.

**Methods:**

We performed a nationwide propensity-score matched (1:1) cohort study using data from the National Health Information Database in the Republic of Korea from 2010 to 2017. The study included 110,723 individuals with diabetes, TG levels ≥ 150 mg/dL, and no prior diagnoses of ASCVD who used statins and fenofibrate, and an equal matched number of similar patients who used statins alone (control group). The study outcomes included newly diagnosed myocardial infarction (MI), stroke, both (MI and/or stroke), and all-cause mortality.

**Results:**

Over a mean 4.03-year follow-up period, the hazard ratios (HR) for outcomes in the fenofibrate group in comparison to the control group were 0.878 [95% confidence interval (CI) 0.827–0.933] for MI, 0.901 (95% CI 0.848–0.957) for stroke, 0.897 (95% CI 0.858–0.937) for MI and/or stroke, and 0.716 (95% CI 0.685–0.749) for all-cause death. These beneficial effects of fenofibrate were consistent in the subgroup with TG 150–199 mg/dL but differed according to low-density lipoprotein cholesterol (LDL-C) levels.

**Conclusion:**

In this nationwide propensity-score matched cohort study involving individuals with diabetes and TG ≥ 150 mg/dL, the risk of all-cause death and ASCVD was significantly lower with fenofibrate use in conjunction with statin treatment compared to statin treatment alone. However, this finding was significant only in individuals with relatively high LDL-C levels.

**Graphical Abstract:**

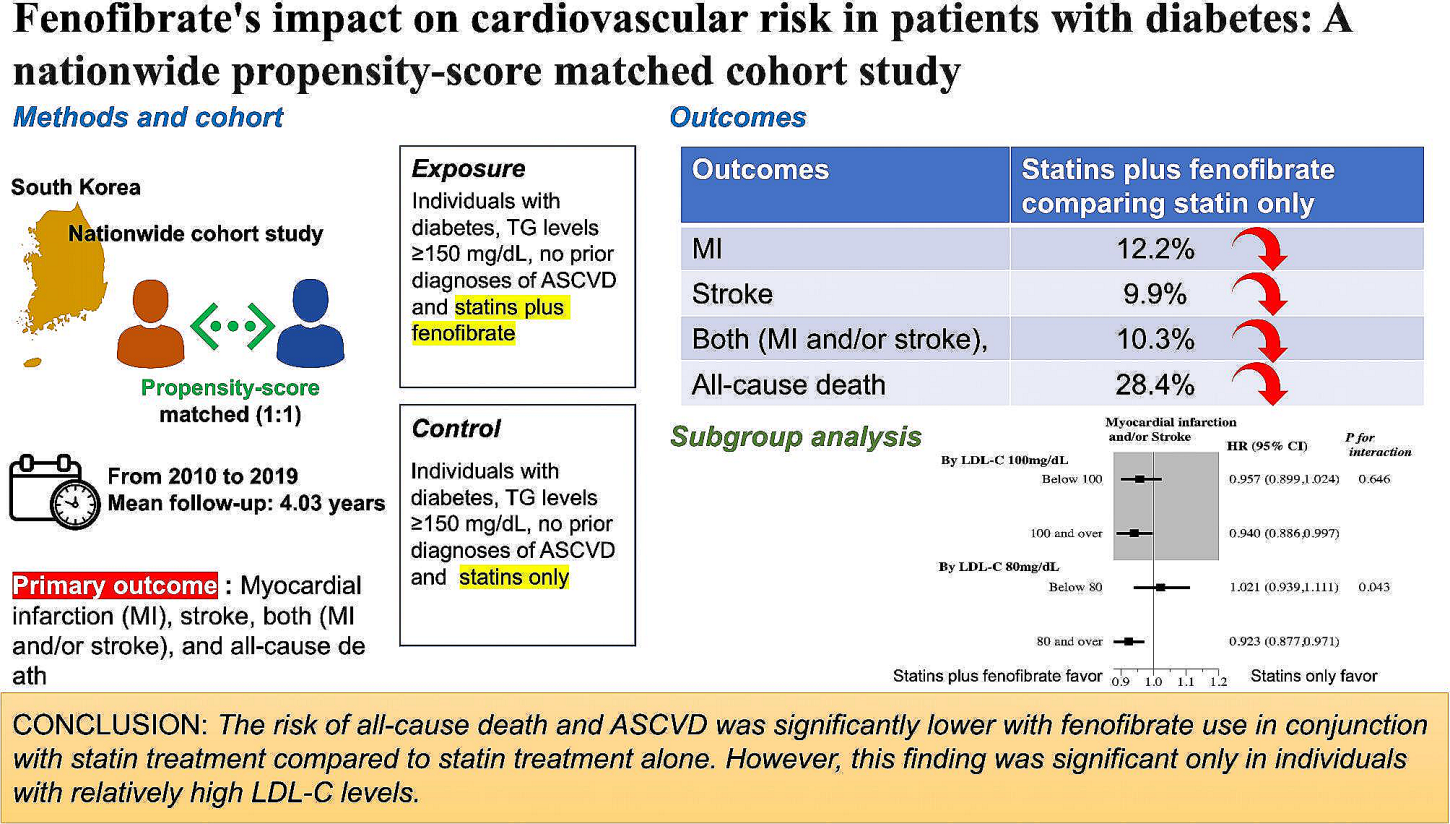

**Supplementary Information:**

The online version contains supplementary material available at 10.1186/s12933-024-02353-5.

## Background

Atherosclerotic cardiovascular disease (ASCVD) is the most prevalent cause of mortality and morbidity in individuals with type 2 diabetes mellitus (T2DM) [1, 2]. Among the major risk factors for ASCVD, dyslipidemia is a key contributor to the increased risk of ASCVD among patients with T2DM [3]. Therefore, most guidelines recommend obtaining a lipid profile at the onset of diabetes diagnosis as part of the initial medical assessment. They recommend implementing lifestyle modifications and statin therapies to mitigate the risk of developing ASCVD in individuals with diabetes [4–7].

Fenofibrate, a peroxisome proliferator-activated receptor-α agonist, is Food and Drug Administration (FDA) approved to reduce levels of elevated low-density lipoprotein-cholesterol (LDL-C), total cholesterol, triglycerides (TG), and apolipoprotein B and to increase levels of high-density lipoprotein-cholesterol (HDL-C) in adult patients with primary hypercholesterolemia or mixed dyslipidemia. However, the efficacy of fenofibrate in comparison to statins on ASCVD outcomes is notably weaker [8, 9]. Therefore, most recent guidelines recommended fenofibrate as the most reliable drug to reduce TG levels in individuals with hypertriglyceridemia [5–7]. Additionally, fenofibrate’s effect on cardiovascular morbidity and mortality in individuals with diabetes is also not clear. Both the Fenofibrate Intervention and Event Lowering in Diabetes (FIELD) and the Action to Control Cardiovascular Risk in Diabetes (ACCORD) trials failed to show a reduction in cardiovascular morbidity and mortality in individuals with diabetes [10, 11]. Only subgroup analyses with participants with elevated TG/low HDL-C levels demonstrated the beneficial effects of fenofibrate on ASCVD outcomes [10–12]. However, a recent study with pemafibrate did not indicate any beneficial effects on ASCVD in individuals with these factors [13]. 

While both promising and unpromising results have emerged in patients with these factors, no large-scale long-term follow-up study has assessed the beneficial effects of fenofibrate on ASCVD outcomes in patients with diabetes already on statin treatment. Hence, we conducted a large, population-based, propensity-score matched cohort study to evaluate the real-world setting efficacy of fenofibrate as an add-on to statin treatment on all-cause death and ASCVD in individuals with diabetes and TG ≥ 150 mg/dL using a large-scale population dataset from the National Health Information Database (NHID).

## Methods

### Study database

The data for our analysis were obtained from the NHID, a public database that encompasses healthcare utilization data for most of the Korean population. This database is linked to the National Death Registry, the National Health Screening Program (NHSP), and the Rare Incurable Disease Registry [14, 15, 16]. The study protocol was approved by the Institutional Review Board of the Kangbuk Samsung Hospital (KBSMC 2021-11-026), which waived the requirement for informed consent due to the unavailability of personal information.

### Study participants

This study included 221,446 participants. We initially identified 1,465,824 patients who were prescribed fenofibrate by the NHID between 2010 and 2017. Of these, we excluded 516,141 individuals who did not receive statin treatment before starting fenofibrate treatment, 335,328 individuals who did not participate in the NHSP two years before starting fenofibrate, 15 individuals below the age of 20 years, 150,784 individuals with TG < 150 mg/dL, 28,379 individuals whose data were incomplete, 298,001 individuals without diabetes, and 23,014 individuals with a history of myocardial infarction (MI) or stroke. After the additional exclusion of 1,357 patients who developed MI or stroke within one year (with a 1-year lag period) of observation, 112,805 patients were included in the analysis. A control group, consisting of 326,894 individuals without fenofibrate treatment, was identified using the same criteria.

**Fig. 1:**
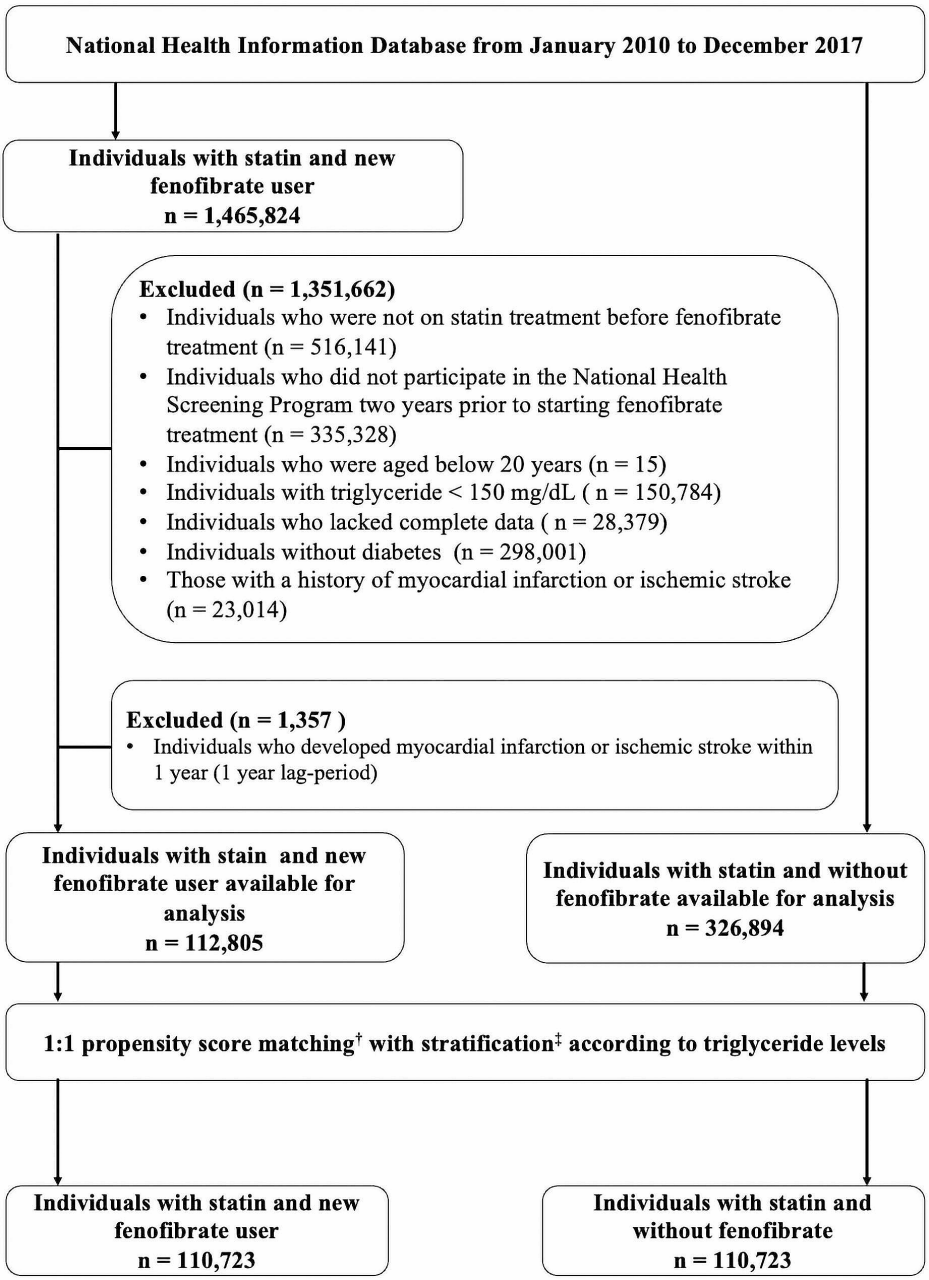
Study population selection flowchart. † Propensity score matching included age, sex, smoking status, alcohol intake, physical activity, body mass index, history of hypertension and chronic kidney disease, fasting glucose, HDL-C and LDL-C levels, number of medications used to treat type 2 diabetes, and simultaneous use of insulin. Stratification has been performed according to TG levels of 200 mg/dL (above or below)

To overcome potential bias due to the difference in baseline characteristics, we performed 1:1 matching with propensity scores. These included variables such as age, sex, smoking status, alcohol intake, physical activity, body mass index (BMI), history of hypertension and chronic kidney disease, fasting glucose, HDL-C and LDL-C levels, number of medications used to treat type 2 diabetes, and simultaneous use of insulin. Additionally, stratification was performed according to TG levels below 200 mg/dL and 200 mg/dL and above (Fig. 1; Table 1, and Table S1). Ultimately, 110,723 patients were included in the fenofibrate group, with an equal number of patients in the control group, matched using propensity scores.

**Table 1:** Baseline characteristics of the study population according to the use of fenofibrate

	Without fenofibrate	With fenofibrate	ASMD^b^
Number of individuals	110,723	110,723	
Age, years	55.71 ± 11.07	55.8 ± 10.5	0.0084
≥65 years old	24,302 (21.95)	22,955 (20.73)	0.0297
Male, %	72,419 (65.41)	72,484 (65.46)	0.0012
Height, cm	164.4 ± 9.27	164.23 ± 9.16	0.0184
Weight, kg	70.93 ± 13	70.75 ± 12.57	0.0137
Body mass index, kg/m^2^	26.13 ± 3.53	26.12 ± 3.35	0.0029
≥25 (Obesity)	67,011 (60.52)	67,993 (61.41)	0.0182
Waist circumference, cm	88.01 ± 8.72	88.04 ± 8.41	0.0037
Current smoker	35,933 (32.45)	35,964 (32.48)	0.0006
Alcohol consumption once or more per week	58,029 (52.41)	57,979 (52.36)	0.0009
Regular physical activity	20,769 (18.76)	20,966 (18.94)	0.0045
Low income	24,481 (22.11)	24,143 (21.8)	0.0074
Systolic blood pressure, mmHg	129.98 ± 15.38	129.72 ± 15.2	0.0169
Diastolic blood pressure, mmHg	80.41 ± 10.26	80.34 ± 10.18	0.0074
Hypertension	67,309 (60.79)	67,018 (60.53)	0.0054
Chronic kidney disease	8917 (8.05)	8875 (8.02)	0.0014
Congestive heart failure	2592 (2.34)	2527 (2.28)	0.0040
Fasting plasma glucose, mg/dL	157.91 ± 54.44	158.01 ± 55.25	0.0017
Total cholesterol, mg/dL	205.58 ± 44.12	210.13 ± 48.28	0.0983
Triglyceride level^a^, mg/dL	251.7 (251.21–252.19)	285.22 (284.53–285.91)	0.3456
<200 mg/dL	23,627	23,627	
≥200 mg/dL	89,178	89,178	
HDL cholesterol, mg/dL	47.01 ± 16.25	46.62 ± 15.26	0.0246
LDL cholesterol, mg/dL	107.83 ± 41.96	107.26 ± 45.19	0.0132
eGFR, ml/min/1.73 m^2^	90.59 ± 48.81	89.83 ± 43.68	0.0163
Number of medications for diabetes			
0	16,969 (15.33)	17,032 (15.38)	
1	18,704 (16.89)	19,753 (17.84)	
2	39,131 (35.34)	38,192 (34.49)	
≥3	35,919 (32.44)	35,746 (32.28)	
Duration of medications for diabetes			
<5 years	52,360 (47.29)	51,858 (46.84)	
≥5 years	41,394 (37.39)	41,833 (37.78)	

^a^ Geometric mean (95% confidence interval)

^b^ ASMD; Absolute Standardized Mean Difference

Estimated glomerular filtration rate; eGFR, high-density lipoprotein cholesterol; HDL-C, low-density lipoprotein cholesterol; LDL-C

### Definitions of diabetes and study outcomes (cardiovascular events and death)

Individuals with type 2 diabetes were identified either through insurance claims data, indicating the prescription of anti-diabetic drugs under the International Classification of Diseases, Tenth Revision (ICD-10) diagnostic codes E11 to E14, or through NHSP records of fasting plasma glucose levels ≥ 126 mg/dL. The outcomes of the study were newly diagnosed MI, stroke, both (MI and/or stroke), and all-cause mortality. Stroke cases were defined as instances with ICD10 codes I63 or I64 during hospitalization lasting more than three days, in conjunction with claims for brain magnetic resonance imaging or brain computed tomography. Meanwhile, MI was defined by ICD10 codes I21 or I22 during hospitalization lasting more than three days, along with claims for percutaneous coronary intervention (PCI) or coronary artery bypass grafting (CABG). The study population was followed from baseline until any of the following events occurred: the date of death, the occurrence of incident ASCVD, or until December 31, 2019, whichever came first.

### Measurements and definitions

All participants completed a questionnaire on their medical history, use of tobacco and alcohol, and exercise habits. Smoking habits were categorized as non-current or current smokers, while alcohol intake was categorized as consumption once or more per week or others. Regular exercise was defined as vigorous exercise three or more times per week or moderate exercise five or more times per week. Low household income was defined as the lowest-income quintile. BMI was calculated as body weight in kilograms divided by the square of height in meters (Obesity = BMI ≥ 25 kg/m^2^). Blood pressure was measured using a standard procedure with a sphygmomanometer after resting for more than 5 min. Blood samples were collected after overnight fasting for more than eight hours. Plasma glucose, total cholesterol, TG, HDL-C, and LDL-C were also measured. We calculated the estimated glomerular filtration rate (eGFR) using the equation from the Modification of Diet in Renal Disease Study: eGFR = 175 × serum creatinine^−1.154^ × age^−0.203^ × 0.742 (for women). Baseline comorbidities were identified as hypertension (blood pressure ≥ 140/90 mmHg or prescription of anti-hypertensive drugs under ICD10 codes I10–I15), chronic kidney disease (eGFR < 60 mL/ min per 1.73 m^2^), and congestive heart failure (I50 as the discharge diagnosis).

### Statistical analyses

To minimize selection bias, we used 1:1 nearest-neighbor propensity score matching (PSM; caliper width, 0.2 standard deviations from the logit propensity score) to select a control group that did not receive fenofibrate. Variables that could potentially affect treatment assignments or outcomes were selected, including sociodemographic characteristics mentioned earlier, comorbidities, and concomitant drugs. The standardized difference was calculated to assess the balance between the two groups, with values < 0.100 considered adequately balanced (Table 1 and Table S1). We also selected a control group stratified based on TG levels of 200 mg/dL and above or below, to minimize selection bias. To minimize reverse causality, we adopted a first-year lag period. Follow-up duration was obtained for each group. Incidence rates were presented as the number of events occurring per 1000 person-years. Hazard ratios (HR) and 95% confidence intervals (CI) for outcomes were calculated using the Cox proportional hazards model. The multivariate models were adjusted for age, sex, smoking status, drinking history, regular physical activity, income, BMI, histories of hypertension, chronic kidney disease, congestive heart failure, fasting glucose, HDL-C and LDL-C levels, eGFR, diabetes duration, the number of medications used to treat diabetes, and the simultaneous use of insulin. Kaplan–Meier survival curves were constructed to compare the incidence rates of the outcomes according to fenofibrate use after adjusting for the aforementioned covariates, and a log-rank test was conducted. Additionally, we performed subgroup analyses according to age, sex, smoking habits, duration of diabetes, histories of hypertension, chronic kidney disease, congestive heart failure, and TG and LDL-C levels. All data analyses were performed using the statistical analysis system version 9.4 (SAS Institute, Cary, NC, USA). Statistical significance was set at *P* < .05.

## Results

### Study population

Before implementing PSM, it was observed that, in comparison to the control group, participants in the fenofibrate group tended to be younger, male, current smokers, and alcohol drinkers, and had DM durations of less than five years. They also had higher TG and LDL-C levels and lower HDL-C levels (Table S1). The baseline characteristics were well balanced between the groups after PSM (all absolute standardized differences were < 0.1, except for TG, as shown in Table 1). In the fenofibrate group, there were 89,178 individuals with TG ≥ 200 mg/dL and 23,627 individuals with TG between 150 and 199 mg/dL. A similar pattern was observed in the control group. The mean ages of the fenofibrate and control groups were 55.7 ± 11.1 and 55.8 ± 10.5 years, respectively.

### Risks of newly diagnosed MI, stroke, MI and/or stroke, and all-cause death

The cumulative prevalence of the outcomes and follow-up durations before and after PSM in the fenofibrate and control groups are shown in Table S 2.

The incidence rates of MI were 4.44 per 1,000 person-years over a mean follow-up period of 4.22 ± 2.16 years in the fenofibrate group and 5.02 per 1,000 person-years in the control group. In the Kaplan–Meier survival analysis for MI, the incidence rate of MI in the fenofibrate group was significantly lower than that in the control group (log-rank, *P* < .001; Figure S1). The risk of MI in the fenofibrate group was 12.2% (95% CI 0.827–0.933; Table 2) lower than that in the control group. The incidence rate of stroke was 4.56 per 1,000 person-years in the fenofibrate group and 5.05 per 1,000 person-years in the control group. In the Kaplan–Meier survival analysis for stroke, the incidence rate of stroke in the fenofibrate group was significantly lower than that in the control group (log-rank test, *P* < .001; Figure S1). The risk for stroke in the fenofibrate group was 9.9% (95% CI 0.848–0.956, Table 2) lower than that in the control group. The incidence rates of MI and/or stroke were 8.68 per 1,000 person-years in the fenofibrate group and 9.63 per 1,000 person-years in the control group. In the Kaplan–Meier survival analysis for MI and/or stroke, the incidence rate of MI and/or stroke in the fenofibrate group was significantly lower than that in the control group (log-rank, *P* < .001, Figure S1). The risk of MI and/or stroke in the fenofibrate group was 10.3% (95% CI, 0.858–0.937; Table 2) lower than that in the control group. The incidence rate of all-cause death was 7.39 per 1,000 person-years in the fenofibrate group and 10.2 per 1,000 person-years in the control group. In the Kaplan–Meier survival analysis for all-cause death, the incidence rate of all-cause death in the fenofibrate group was significantly lower than that in the control group (log-rank test, *P* < .001, Figure S1). The risk of all-cause death in the fenofibrate group was 28.4% (95% CI 0.685–0.749; Table 2) lower than that in the control group.

**Table 2:** Number, incidence rates, and hazard ratios of outcomes in the fenofibrate group and the 1:1 propensity score-matched control group without fenofibrate

	Number of patients	Number of events	Duration (person-years)	Rate*	Hazard ratio (95% confidence interval)
**Myocardial infarction**					
Without fenofibrate	110,723	2135	425,262	5.02	1 (Ref.)
With fenofibrate	110,723	2076	467,220	4.44	0.878 (0.827,0.933)
**Stroke**					
Without fenofibrate	110,723	2141	424,358	5.05	1 (Ref.)
With fenofibrate	110,723	2128	466,288	4.56	0.901 (0.848,0.957)
**Myocardial infarction and/or stroke**					
Without fenofibrate	110,723	4049	420,492	9.63	1 (Ref.)
With fenofibrate	110,723	4014	462,312	8.68	0.897 (0.858,0.937)
**All-cause death**					
Without fenofibrate	110,723	4376	429,485	10.2	1 (Ref.)
With fenofibrate	110,723	3485	471,507	7.39	0.716 (0.685,0.749)

* Events per 1000 person-years

### Subgroup analyses

**Fig. 2:**
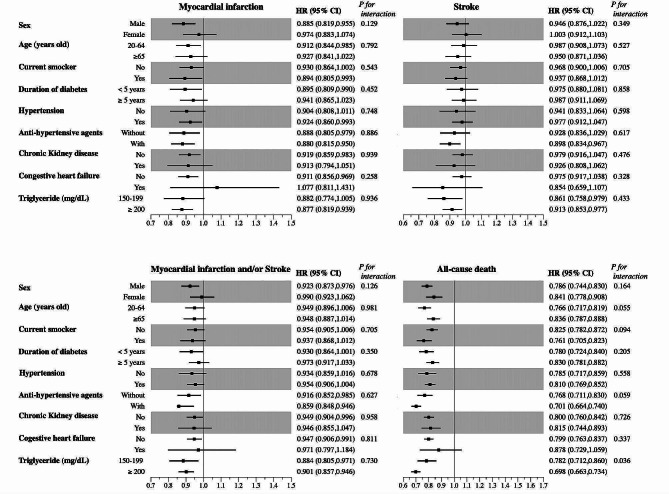
Hazard ratios for outcomes between the fenofibrate group and the 1:1 propensity score-matched control group. Subjects are organized into pre-specified subgroups adjusted for age, sex, smoking status, drinking history, regular physical activity, income, BMI, histories of hypertension, chronic kidney disease, or congestive heart failure, levels of fasting glucose, HDL-C, LDL-C, and eGFR, diabetes duration, number of medications used to treat diabetes, and simultaneous usage of insulin

**Fig. 3:**
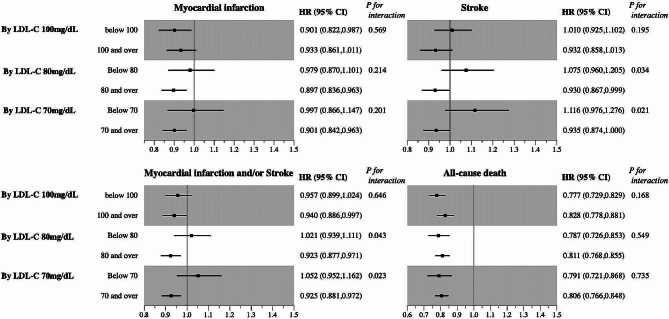
Hazard ratios for outcomes between the fenofibrate group and the 1:1 propensity score-matched control group. The groups are matched according to LDL-C levels and adjusted for age, sex, smoking status, drinking history, regular physical activity, income, BMI, histories of hypertension, chronic kidney disease, or congestive heart failure, levels of fasting glucose, HDL-C, LDL-C, and eGFR, diabetes duration, number of medications used to treat diabetes, and simultaneous usage of insulin

Figure 2 shows the results of the subgroup analyses based on baseline characteristics. No significant interactions were observed between the fenofibrate group and the control group (*P* > .05 for all interactions), except in the subgroup analysis based on TG levels, specifically for all-cause death. Treatment with fenofibrate was associated with a decreased risk of all-cause death at higher TG levels compared to the control group (P for interaction = 0.036). Figure 3 depicts the results of the subgroup analyses based on LDL-C levels. The lower risk of MI in the fenofibrate group compared to the control group was attenuated in the subgroups with lower LDL-C levels (Fig. 3). Moreover, a lower risk of stroke was not observed between the fenofibrate group and control groups in the lower LDL-C level subgroups (< 80 mg/dL and < 70 mg/dL, Fig. 3). However, treatment with fenofibrate was associated with a lower risk of all-cause death across all LDL-C levels (Fig. 3).

### Supplemental analyses

We conducted several sensitivity analyses. Firstly, we analyzed the unadjusted cohort, which consisted of the fenofibrate group (*n* = 112,805) and the control group (*n* = 326,894). This analysis involved a multivariable-adjusted Cox proportional hazards model adjusted for age, sex, smoking status, drinking history, regular physical activity, income, BMI, histories of hypertension, chronic kidney disease, congestive heart failure, fasting glucose, HDL-C and LDL-C levels, eGFR, diabetes duration, the number of medications used to treat diabetes, and the simultaneous use of insulin. The results showed consistent findings that fenofibrate was associated with lower risk of ASCVD and all-cause death (Table S3). Secondly, we analyzed the cohort after PSM, which included the variables mentioned above as well as TG levels (fenofibrate group, *n* = 50,170; control group, *n* = 50,170). Notably, these results remained consistent (Tables S4 and S5). Thirdly, to minimize potential reverse correlations, individuals with pre-existing conditions were excluded, and a three-year lag time was employed. These results remained consistent (Table S6).

## Discussion

This retrospective, nationwide, propensity-score matched cohort study showed that fenofibrate treatment in individuals with diabetes and TG ≥ 150 mg/dL, who were concurrently undergoing statin treatment, was associated with a lower risk of all-cause death or ASCVD compared to matched individuals treated without fenofibrate. This association was observed in all subgroups, even in the subgroup with TG levels between 150 and 199 mg/dL, and the results remained consistent even after various sensitivity analyses. However, the association between a lower risk of ASCVD and fenofibrate was observed only in patients with high LDL-C levels (> 80 mg/dL). Our study provides new insights into mitigating cardiovascular complications in patients with diabetes.

LDL-C is a known causal factor for ASCVD, [17, 18] whereas the role of TG in ASCVD causation remains debated [19]. Although several studies conducted recently, including observational studies, [20–22] a post hoc analysis of a trial, [23] and Mendelian randomization studies, [24–26] have reported a potential link between TG and ASCVD, the impact of therapies aimed at reducing TG levels on ASCVD incidence remains uncertain. A recent clinical trial employing high-dose n-3 fatty acids failed to demonstrate a reduction in ASCVD incidence despite a 20% TG level decrease [27]. Another trial with icosapent ethyl reported ASCVD reductions, but these were not associated with changes in TG levels [28]. Furthermore, additional meta-analyses have indicated that this clinical benefit exceeds what can be solely attributed to triglyceride reduction, suggesting that icosapent ethyl may possess additional pleiotropic effects in reducing ASCVD risk, including anti-inflammatory and anti-aggregatory mechanisms [29, 30]. Similarly, a trial using niacin did not significantly decrease ASCVD risk, even with a 26% reduction in TG levels compared to the placebo group [31]. While previous trials of fenofibrate (FIELD and ACCORD trials) showed no significant decrease in ASCVD risk with TG reductions of 29 and 26%, respectively, [10, 32] subgroup analyses have strongly suggested that patients with elevated TG/low HDL-C levels particularly those with diabetes, may benefit from TG reduction with fenofibrate [10–12]. The anti-atherogenic effects of fenofibrates primarily reduces the secretion of triglyceride-rich very low-density lipoprotein particles by enhancing fatty acid oxidation and reducing hepatic lipogenesis and also modestly reduce apoB levels [33, 34]. Fenofibrate significantly contributes to protecting against metabolic disorders by improving insulin resistance, which is closely linked to irisin resistance and metabolic dysregulation [35]. Additionally, it inhibits systemic inflammatory responses, thereby mitigating ASCVD risk [36]. Like the findings mentioned earlier, our study demonstrated that fenofibrate was associated with lower risks of ASCVD and all-cause death (Table 2). These associations were also observed in the subgroup with TG levels between 150 and 199 mg/dL (Fig. 2). Our findings align with a prior NHID study, which reported that adding fenofibrate to statin therapy was associated with a lower risk of all-cause mortality and CVD in the general population with elevated TG (median TG concentration: 285.25 mg/dL in fenofibrate users and 215.43 mg/dL in fenofibrate non-users) [37] and in patients with metabolic syndrome receiving statin treatment alone (mean TG level: 254 mg/dL in the statin + fenofibrate group and 211 mg/dL in the statins alone group) [38]. Notably, these findings remained consistent across various sensitivity analyses that included propensity scores with TG levels (median TG level: 237 mg/dL in the fenofibrate group and 231 mg/dL in the control group; Tables S4 and S5).

In contrast to our findings, in a recent large placebo-controlled trial (over 10,000 participants) of pemafibrate in patients with type 2 diabetes, a TG level between 200 and 499 mg/dL and an HDL-C level ≤ 40 mg/dL did not reduce the risk of ASCVD, despite achieving TG levels that were 26.2% lower in comparison to placebo [13]. Several factors may have contributed to this discrepancy, such as differences in baseline LDL levels (79 mg/dL vs. 107 mg/dL in our study), the incidence of ASCVD (36 per 1000 person-years vs. 8.60 per 1000 person-years in our study), or distinctions between fenofibrate and pemafibrate. However, like the above pemafibrate study, our study showed that the association between a lower risk of ASCVD and fenofibrate weakened and disappeared in the subgroups with lower LDL-C levels (< 80 mg/dL, Fig. 3). The diminished statistically significant impact of fibrate therapy on ASCVD risk among patients with low LDL-C levels may be associated with the presence of high-intensity statin, in contrast to the ACCORD trial which utilized moderate or low-intensity statins [10]. Conversely, the observed benefit in patients with higher LDL levels may be attributed to the modest impact of fibrate therapy on reducing LDL-C. And this finding also supported by study with pemafibrate, which demonstrated its failure to reduce ASCVD risk and its tendency to increase LDL cholesterol levels in patients with type 2 diabetes, mild-to-moderate hypertriglyceridemia, a low HDL cholesterol level [13]. This indicates that a TG level ≥ 150 mg/dL serves as an indicator of residual risk in patients with diabetes and LDL-C levels ≥ 80 mg/dL who are undergoing statin treatment. In these patients, intensifying statin therapy may be necessary, and considering fenofibrate supplementation could be considered particularly if statin intensification is not an option.

The strength of our study lies in the fact that we used a large-scale nationwide database representing the entire Korean population along with PSM. However, this study has certain limitations. First, as this was a retrospective and observational study, selection bias was unavoidable. To mitigate this, we employed PSM by incorporating confounding factors, stratified the data according to TG levels, performed sensitivity analyses, and used a multivariable-adjusted Cox proportional hazards model. Second, we could not evaluate post-fenofibrate treatment TG levels due to data limitations, and follow-up data for other biomarkers, including various lipid parameters and glucose levels, were similarly unavailable. Third, we defined MI, stroke, and comorbidities such as diabetes, hypertension, and congestive heart failure using claims data. While this method may not be perfectly accurate, we enhanced precision by creating operational definitions that combined diagnosis, blood glucose, blood pressure, and prescription records. Fourth, we did not report the safety of the use of fenofibrate and statins such, as changes in laboratory parameters, including serum AST, ALT, CK, and creatinine levels, and incidence of myopathies in this study due to limitation of our study database. However, the AST, ALT, and creatinine levels between the control group and the fenofibrate group after treatment were not different (Table S7). Fifth, owing to the retrospective nature of this study, causality could not be inferred. However, to minimize the likelihood of reverse causation, we excluded individuals with a history of MI or stroke and adopted a one-year lag period. Finally, the study’s generalizability to other ethnicities may be limited since it focused on the Korean NHID.

## Conclusions

This nationwide propensity score-matched cohort study of patients with diabetes and TG levels ≥ 150 mg/dL showed that fenofibrate, as an add-on to statin treatment, was associated with a lower risk of all-cause mortality and ASCVD. These beneficial effects of fenofibrate were consistent across subgroups, including those with TG levels between 150 and 199 mg/dL. However, this effect was dependent on LDL-C levels. This indicates that a TG level ≥ 150 mg/dL serves as an indicator of residual risk in patients with diabetes and LDL-C levels ≥ 80 mg/dL who are undergoing statin treatment. In these patients, intensifying statin therapy may be necessary, and considering fenofibrate supplementation could be considered particularly if statin intensification is not an option.

### Electronic supplementary material

Below is the link to the electronic supplementary material.

Supplementary Material 1

## Data Availability

The data that support the findings of this study are available from the National Health Insurance Sharing Service (NHISS, https://nhiss.nhis.or.kr/). However, restrictions apply regarding the availability of the data, which were used with permission for the present study, and are therefore not publicly available. However, they may be made available through the corresponding author, upon reasonable request and with permission from the NHISS.

## Abbreviations

ACCORD: Action to Control Cardiovascular Risk in Diabetes
ASCVD: Atherosclerotic cardiovascular disease
BMI: Body mass index
CABG: Coronary artery bypass grafting
CI: Confidence intervals
eGFR: Estimated glomerular filtration rate
FDA: Food and Drug Administration
FIELD: Fenofibrate Intervention and Event Lowering in Diabetes
HDL-C: High-density lipoprotein-cholesterol
HR: Hazard ratios
ICD: International Classification of Diseases
LDL-C: Low-density lipoprotein-cholesterol
MI: Myocardial infarction
NHID: National Health Information Database
NHSP: National Health Screening Program
PCI: Percutaneous coronary intervention
PSM: Propensity score matching
T2DM: Type 2 diabetes mellitus
TG: Triglycerides
