# Supplementary material for: Fenofibrate’s impact on cardiovascular risk in patients with diabetes: a nationwide propensity-score matched cohort study
Source: Cardiovasc Diabetol. 2024 Jul 18;23:263. doi: 10.1186/s12933-024-02353-5 (PMC11264858; doi:10.1186/s12933-024-02353-5)
Supplement: Supplementary file 1 — Supplementary Material 1 [file 12933_2024_2353_MOESM1_ESM.docx]

Supplementary table 1. Baseline characteristics of the study population according to the use of fenofibrate before and after 1:1 matching with propensity score and stratification by triglyceride levels.

|  | Before propensity score matching | | | After propensity score matching | | |
| --- | --- | --- | --- | --- | --- | --- |
|  | Without fenofibrate | With fenofibrate | ASMD^‡^ | Without fenofibrate | With fenofibrate | ASMD^‡^ |
| Number of individuals | 326894 | 112805 |  | 110723 | 110723 |  |
| Age, years | 58.28±11.31 | 55.57±10.59 | 0.2478 | 55.71±11.07 | 55.8±10.5 | 0.0084 |
| ≥65 years old | 97806(29.92) | 22963(20.36) | 0.2218 | 24302(21.95) | 22955(20.73) | 0.0297 |
| Male, % | 193030(59.05) | 74373(65.93) | 0.1425 | 72419(65.41) | 72484(65.46) | 0.0012 |
| Height, cm | 162.94±9.41 | 164.36±9.17 | 0.1521 | 164.4±9.27 | 164.23±9.16 | 0.0184 |
| Weight, kg | 69.06±12.59 | 70.95±12.68 | 0.1490 | 70.93±13 | 70.75±12.57 | 0.0137 |
| Body mass index, kg/m^2^ | 25.9±3.46 | 26.15±3.36 | 0.0717 | 26.13±3.53 | 26.12±3.35 | 0.0029 |
| ≥ 25 (Obesity) | 189784(58.06) | 69568(61.67) | 0.0738 | 67011(60.52) | 67993(61.41) | 0.0182 |
| Waist circumference, cm | 87.38±8.61 | 88.11±8.45 | 0.0853 | 88.01±8.72 | 88.04±8.41 | 0.0037 |
| Current smoker | 86330(26.41) | 37298(33.06) | 0.1460 | 35933(32.45) | 35964(32.48) | 0.0006 |
| Alcohol once or more per week | 147418(45.1) | 59778(52.99) | 0.1584 | 58029(52.41) | 57979(52.36) | 0.0009 |
| Regular physical activity | 62552(19.14) | 21307(18.89) | 0.0063 | 20769(18.76) | 20966(18.94) | 0.0045 |
| Low income | 68856(21.06) | 24560(21.77) | 0.0173 | 24481(22.11) | 24143(21.8) | 0.0074 |
| Systolic blood pressure, mm Hg | 129.98±15.46 | 129.74±15.2 | 0.0155 | 129.98±15.38 | 129.72±15.2 | 0.0169 |
| Diastolic blood pressure, mm Hg | 79.87±10.16 | 80.39±10.19 | 0.0512 | 80.41±10.26 | 80.34±10.18 | 0.0074 |
| Hypertension | 194313(59.44) | 68442(60.67) | 0.0251 | 67309(60.79) | 67018(60.53) | 0.0054 |
| Chronic kidney disease | 31792(9.73) | 8926(7.91) | 0.0640 | 8917(8.05) | 8875(8.02) | 0.0014 |
| Congestive heart failure | 7761(2.37) | 2570(2.28) | 0.0060 | 2592(2.34) | 2527(2.28) | 0.0040 |
| Fasting plasma glucose, mg/dL | 156.16±53.79 | 157.94±55.15 | 0.0327 | 157.91±54.44 | 158.01±55.25 | 0.0017 |
| Total cholesterol, mg/dL | 210.73±45.64 | 209.71±48.39 | 0.0218 | 205.58±44.12 | 210.13±48.28 | 0.0983 |
| Triglyceride^†^, mg/dL | 220.33(220.09-220.57) | 287.41(286.71-288.1) | 0.6463 | 251.7(251.21-252.19) | 285.22(284.53-285.91) | 0.3456 |
| HDL cholesterol, mg/dL | 48.48±15.77 | 46.51±15.2 | 0.1274 | 47.01±16.25 | 46.62±15.26 | 0.0246 |
| LDL cholesterol, mg/dL | 117.49±42.9 | 106.36±45.47 | 0.2518 | 107.83±41.96 | 107.26±45.19 | 0.0132 |
| eGFR, ml/min/1.73 m^2^ | 88.46±46.19 | 90.02±43.85 | 0.0346 | 90.59±48.81 | 89.83±43.68 | 0.0163 |
| Numbers of medications for diabetes | |  |  |  |  |  |
| 0 | 87774(26.85) | 17033(15.1) |  | 16969(15.33) | 17032(15.38) |  |
| 1 | 54479(16.67) | 19948(17.68) |  | 18704(16.89) | 19753(17.84) |  |
| 2 | 100367(30.7) | 38983(34.56) |  | 39131(35.34) | 38192(34.49) |  |
| ≥3 | 84274(25.78) | 36841(32.66) |  | 35919(32.44) | 35746(32.28) |  |
| Duration of medications for diabetes | |  |  |  |  |  |
| < 5 years | 114830(35.13) | 53757(47.65) |  | 52360(47.29) | 51858(46.84) |  |
| ≥ 5 years | 124290(38.02) | 42015(37.25) |  | 41394(37.39) | 41833(37.78) |  |

† Geometric Mean (95% confidence interval)

‡ ASMD; Absolute Standardized Mean Difference

Supplementary table 2. The cumulative prevalence of outcomes and follow up duration in the fenofibrate and control groups.

|  | Before propensity score matching | | After propensity score matching | |
| --- | --- | --- | --- | --- |
|  | Without fenofibrate | With fenofibrate | Without fenofibrate | With fenofibrate |
| Number of individuals | 326894 | 112805 | 110723 | 110723 |
| Outcome |  |  |  |  |
| Myocardial infarction | 6690(2.05) | 2103(1.86) | 2135(1.93) | 2076(1.87) |
| Stroke | 7219(2.21) | 2145(1.9) | 2141(1.93) | 2128(1.92) |
| Myocardial infarction and stroke | 13181(4.03) | 4055(3.59) | 4049(3.66) | 4014(3.63) |
| all-caused death | 14636(4.48) | 3533(3.13) | 4376(3.95) | 3485(3.15) |
| Follow up duration, Mean±SD | |  |  |  |
| MI | 3.84±2.23 | 4.22±2.16 | 3.84±2.23 | 4.22±2.16 |
| STROKE | 3.82±2.23 | 4.22±2.16 | 3.83±2.23 | 4.21±2.16 |
| CVD | 3.79±2.22 | 4.18±2.17 | 3.8±2.22 | 4.18±2.16 |
| DEATH | 3.88±2.23 | 4.26±2.16 | 3.88±2.23 | 4.26±2.16 |
| F/U duration, Median (Q1-Q3) | |  |  |  |
| MI | 3.43 (1.83-5.5) | 4.04 (2.33-5.85) | 3.44 (1.84-5.5) | 4.04 (2.33-5.84) |
| STROKE | 3.42 (1.82-5.49) | 4.03 (2.33-5.84) | 3.42 (1.83-5.49) | 4.03 (2.33-5.83) |
| CVD | 3.36 (1.8-5.44) | 3.99 (2.28-5.8) | 3.38 (1.81-5.44) | 3.99 (2.28-5.8) |
| DEATH | 3.48 (1.85-5.55) | 4.09 (2.37-5.89) | 3.48 (1.86-5.55) | 4.08 (2.37-5.88) |

Supplementary table 3. Number, incidence rate, and hazard ratio of outcomes in the fenofibrate group and the control group without fenofibrate before PSM.

|  | Number of patients | Number of events | Duration  (person-years) | Rate* | Hazard ratio (95% confidence interval) | | | |
| --- | --- | --- | --- | --- | --- | --- | --- | --- |
|  |  |  |  |  | Unadjusted | Model 1 | Model 2 | Model 3 |
| **Myocardial infarction** | |  |  |  |  |  |  |  |
| No | 326894 | 6690 | 1253883 | 5.34 | 1(Ref.) | 1(Ref.) | 1(Ref.) | 1(Ref.) |
| Yes | 112805 | 2103 | 476464 | 4.41 | 0.821(0.782,0.862) | 0.947(0.901,0.996) | 0.962(0.915,1.011) | 0.946(0.900,0.995) |
| **Stroke** |  |  |  |  |  |  |  |  |
| No | 326894 | 7219 | 1250322 | 5.77 | 1(Ref.) | 1(Ref.) | 1(Ref.) | 1(Ref.) |
| Yes | 112805 | 2145 | 475544 | 4.51 | 0.778(0.741,0.817) | 0.967(0.921,1.015) | 0.979(0.932,1.028) | 0.968(0.922,1.017) |
| **Myocardial infarction and/or stroke** | | |  |  |  |  |  |  |
| No | 326894 | 13181 | 1238259 | 10.6 | 1(Ref.) | 1(Ref.) | 1(Ref.) | 1(Ref.) |
| Yes | 112805 | 4055 | 471516 | 8.60 | 0.804(0.776,0.833) | 0.959(0.925,0.994) | 0.974(0.940,1.010) | 0.961(0.927,0.997) |
| **All-cause death** | |  |  |  |  |  |  |  |
| No | 326894 | 14636 | 1267054 | 11.6 | 1(Ref.) | 1(Ref.) | 1(Ref.) | 1(Ref.) |
| Yes | 112805 | 3533 | 480809 | 7.35 | 0.627(0.605,0.651) | 0.859(0.827,0.891) | 0.862(0.831,0.895) | 0.850(0.819,0.883) |

* Events per 1000 person-years

Model 1; age, sex, smoking status, drinking history, regular physical activity, income, BMI, a history of hypertension, chronic kidney disease, and congestive heart failure.

Model 2; age, sex, smoking status, drinking history, regular physical activity, income, BMI, a history of hypertension, chronic kidney disease, and congestive heart failure, a level of fasting glucose, HDL-C, LDL-C, and eGFR, and LDL.

Model 3; age, sex, smoking status, drinking history, regular physical activity, income, BMI, a history of hypertension, chronic kidney disease, and congestive heart failure, a level of fasting glucose, HDL-C, LDL-C, and eGFR, and LDL, diabetes duration, numbers of medications used to treat diabetes, and simultaneous usage of insulin.

Supplementary table 4. Baseline characteristics of the study population according to the use of fenofibrate after 1:1 matching with propensity score*

|  | Without fenofibrate | With fenofibrate | ASMD**^‡^** |
| --- | --- | --- | --- |
| Number of individuals | 50170 | 50170 |  |
| Age, years | 56.62±10.31 | 56.54±10.41 | 0.0079 |
| ≥65 years old | 11571(23.06) | 11436(22.79) | 0.0064 |
| Male, % | 31606(63) | 32025(63.83) | 0.0173 |
| Height, cm | 163.83±9.24 | 163.87±9.21 | 0.0050 |
| Weight, kg | 70.14±12.51 | 70.17±12.46 | 0.0024 |
| Body mass index, kg/m^2^ | 26.03±3.43 | 26.02±3.35 | 0.0032 |
| ≥ 25 (Obesity) | 29964(59.72) | 30222(60.24) | 0.0105 |
| Waist circumference, cm | 87.68±8.55 | 87.75±8.44 | 0.0085 |
| Current smoker | 14859(29.62) | 14976(29.85) | 0.0051 |
| Alcohol once or more per week | 24603(49.04) | 24794(49.42) | 0.0076 |
| Regular physical activity | 9721(19.38) | 9739(19.41) | 0.0009 |
| Low income | 10586(21.1) | 10506(20.94) | 0.0039 |
| Systolic blood pressure, mm Hg | 129.61±15.24 | 129.44±15.2 | 0.0109 |
| Diastolic blood pressure, mm Hg | 79.95±10.12 | 79.99±10.12 | 0.0042 |
| Hypertension | 30401(60.6) | 30299(60.39) | 0.0042 |
| Chronic kidney disease | 4224(8.42) | 4227(8.43) | 0.0002 |
| Fasting plasma glucose, mg/dL | 155.13±53.15 | 156.29±54.68 | 0.0214 |
| Total cholesterol, mg/dL | 207.9±46.8 | 205.61±46.63 | 0.0491 |
| Triglyceride**^†^**, mg/dL | 230.91(230.24-231.58) | 236.63(235.98-237.29) | 0.0456 |
| HDL cholesterol, mg/dL | 47.88±15.15 | 47.92±16.89 | 0.0028 |
| LDL cholesterol, mg/dL | 113.21±44.07 | 109.78±43.38 | 0.0784 |
| eGFR, ml/min/1.73 m^2^ | 89.39±46.37 | 89.02±41.59 | 0.0083 |
| Numbers of medications for diabetes | |  |  |
| 0 | 8616(17.17) | 9576(19.09) |  |
| 1 | 8822(17.58) | 8949(17.84) |  |
| 2 | 17456(34.79) | 16704(33.29) |  |
| ≥3 | 15276(30.45) | 14941(29.78) |  |
| Duration of medications for diabetes | |  |  |
| < 5 years | 21063(41.98) | 20217(40.3) |  |
| ≥ 5 years | 20491(40.84) | 20377(40.62) |  |

* propensity score included age, sex, smoking status, alcohol intake, physical activity, body mass index (BMI), a history of hypertension and chronic kidney disease, a level of fasting glucose, HDL-C, triglyceride, and LDL-C, numbers of medications used to treat type 2 diabetes, and simultaneous usage of insulin

Supplementary table 5. Number, incidence rate, and hazard ratio of outcomes in the fenofibrate group and the 1:1 propensity score mating* control group without fenofibrate.

|  | Number of patients | Number of events | Duration  (person-years) | Rate* | Hazard ratio  (95% confidence interval) |
| --- | --- | --- | --- | --- | --- |
| **Myocardial infarction** | | |  |  |  |
| No | 50170 | 1019 | 206066 | 4.95 | 1(Ref.) |
| Yes | 50170 | 908 | 208830 | 4.35 | 0.879(0.804,0.961) |
| **Stroke** |  |  |  |  |  |
| No | 50170 | 1009 | 205762 | 4.90 | 1(Ref.) |
| Yes | 50170 | 964 | 208489 | 4.62 | 0.942(0.863,1.029) |
| **Myocardial infarction and/or stroke** | | | |  |  |
| No | 50170 | 1911 | 203914 | 9.37 | 1(Ref.) |
| Yes | 50170 | 1788 | 206687 | 8.65 | 0.923(0.865,0.984) |
| **All-cause death** | | |  |  |  |
| No | 50170 | 1898 | 208098 | 9.12 | 1(Ref.) |
| Yes | 50170 | 1584 | 210752 | 7.52 | 0.824(0.771,0.881) |

* propensity score included age, sex, smoking status, alcohol intake, physical activity, body mass index (BMI), a history of hypertension and chronic kidney disease, a level of fasting glucose, HDL-C, triglyceride, and LDL-C, numbers of medications used to treat type 2 diabetes, and simultaneous usage of insulin

Supplementary table 6. Number, incidence rate, and hazard ratio of outcomes in the fenofibrate group and the 1:1 propensity score mating control group without fenofibrate with a 3-year lag time.

|  | Number of patients | Number of events | Duration  (person-years) | Rate* | Hazard ratio  (95% confidence interval) |
| --- | --- | --- | --- | --- | --- |
| **Myocardial infarction** | | | |  |  |
| No | 78741 | 1258 | 220512 | 5.71 | 1(Ref.) |
| Yes | 88113 | 1213 | 255822 | 4.74 | 0.831(0.768,0.899) |
| **Stroke** |  |  |  |  |  |
| No | 78741 | 1169 | 62833 | 18.6 | 1(Ref.) |
| Yes | 88113 | 1241 | 79158 | 15.7 | 0.910(0.810,1.023) |
| **Myocardial infarction and/or stroke** | | | |  |  |
| No | 78741 | 2306 | 375891 | 6.14 | 1(Ref.) |
| Yes | 88113 | 2349 | 429779 | 5.47 | 0.877(0.828,0.929) |
| **All-cause death** | |  |  |  |  |
| No | 78741 | 2607 | 222559 | 11.7 | 1(Ref.) |
| Yes | 88113 | 2184 | 257796 | 8.47 | 0.724(0.684,0.766) |

Supplementary table 7. AST, ALT, and creatinine levels of the study population according to the use of fenofibrate after treatment.

|  | Without fenofibrate | With fenofibrate | ASMD |
| --- | --- | --- | --- |
| n | 110723 | 110723 |  |
| HE_CREA | 0.95±0.67 | 0.94±0.7 | 0.0039 |
| *AST | 28.06(27.98-28.14) | 28.9(28.81-28.98) | 0.0451 |
| *ALT | 31.19(31.08-31.3) | 32.04(31.93-32.15) | 0.0263 |
| *rGTP | 48.7(48.47-48.92) | 53.72(53.46-53.98) | 0.0946 |

***Geometric Mean (95% confidence interval)**
